# Au and Ti closer in TS-1 zeolite for enhancing activity

**DOI:** 10.1093/nsr/nwag310

**Published:** 2026-05-30

**Authors:** Zi-Chen Zheng, Li-Hua Chen, Bao-Lian Su

**Affiliations:** Chimie des Interactions Plasma-Surface, University of Mons, Belgium; Laboratory of Inorganic Materials Chemistry (CMI), University of Namur, Belgium; State Key Laboratory of Advanced Technology for Materials Synthesis and Processing, Wuhan University of Technology, China; Laboratory of Inorganic Materials Chemistry (CMI), University of Namur, Belgium; State Key Laboratory of Advanced Technology for Materials Synthesis and Processing, Wuhan University of Technology, China

Hierarchical porosity has long been used to improve diffusion in zeolites, but its catalytic value depends on more than simply creating extra void space. Earlier work already showed that mesopore quality and connectivity, rather than pore volume alone, can strongly affect accessibility and catalyst lifetime [1]. More recent studies on hierarchical zeolite synthesis further emphasized that pore morphology must be designed together with crystal architecture [2]. Specifically, TS-1 is a support that has been extensively studied in selective oxidation and propylene epoxidation. To enhance its catalytic performance, it is not only about improving the porosity of the TS-1 zeolite support, but also about constructing an intracrystalline framework that can modulate the spatial relationship between framework Ti sites and deposited metal species [3,4].

Take the direct epoxidation of propylene with H_2_ and O_2_ over Au/TS-1 as an example. In this tandem reaction, Au sites are responsible for H_2_O_2_ synthesis from H_2_+O_2_ reaction, whereas framework Ti sites mediate olefin epoxidation. Efficient catalysis requires these two functional sites to work in close proximity [5]. Earlier studies therefore tried to manipulate where Au resides in TS-1 crystals [5], to improve catalyst preparation and stability by tuning the charge state [6], to engineer sodium-decorated Au−Ti sites that accelerate molecular transfer [4] and to regulate TS-1 surface properties and morphology [7]. Yet these advances still operated largely within the transport constraints of the microporous Mobil-type Five (MFI) framework.

Liu *et al.* reported a gold catalyst stabilized in an open-porosity TS-1 zeolite, which addresses this problem at the level of intracrystalline architecture rather than by incremental surface modification alone, and their design concept was displayed in Fig. 1a [8]. Their strategy combines polyethylenimine with a dual-functional silane linkage reagent to direct zeolite growth around polymer chains, generating open channels across the crystallites after template removal. Compared with the conventional Au/TS-NaOH catalyst prepared via the conventional etching treatment with NaOH solution, the Au/TS-GPI catalyst based on open-porosity TS-1 zeolite exhibits a striking redistribution of Au species from the outer regions into the zeolite interior, as shown in Fig. 1(b–i). The Au nanoclusters are much more uniformly dispersed throughout the open-porosity TS-1 crystals, which increases the abundance of neighboring Au-Ti site pairs needed for direct propylene epoxidation. That spatial reorganization translates into strong performance gains. The open-porosity catalyst delivers nearly 3-fold higher specific mass activity than the conventional reference while maintaining similarly high propylene oxide selectivity and H_2_ utilization efficiency. It also shows improved stability and extends the reaction scope to larger olefins such as 1-hexene, cyclohexene and 1-octene. This broader substrate scope is consistent with the general view that pore architecture can regulate accessibility under diffusion-limited conditions [1]. More importantly, this study shows that the open-porosity zeolite structure enables the formation of intimately contacted bifunctional sites within the zeolite matrix, which hold great promise in tandem catalytic reactions.

**Figure 1.**
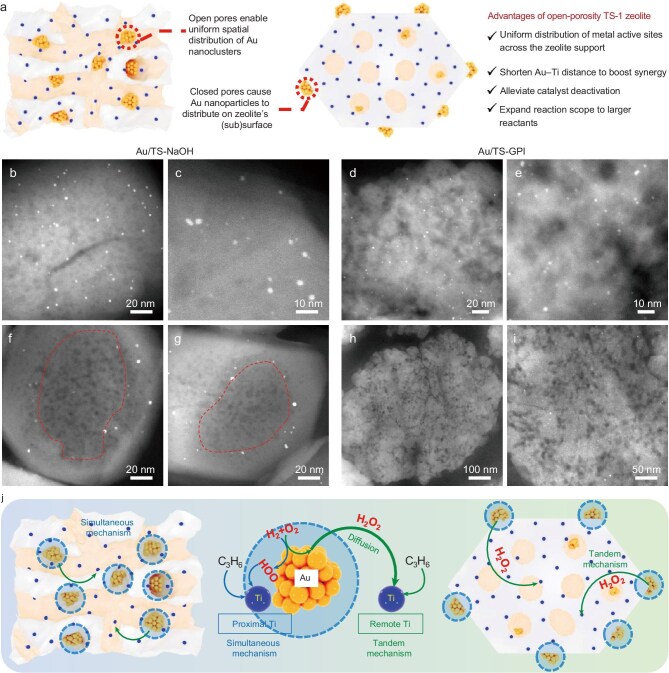
(a) Concept of the open-porosity catalyst design. (b−e) High-angle annular dark-field scanning transmission electron microscopy (HAADF-STEM) images of Au/TS-NaOH (b and c) and Au/TS-GPI (d and e) samples. Gold particles supported on the zeolite supports are visualized as bright particles in these images. (f−i) HAADF-STEM images of 70-nm-thick Au/TS-NaOH (f and g) and Au/TS-GPI (h and i) samples. (j) Schematic illustration of the proximity-enabled reaction picture. Adapted with permission from Liu *et al*. [8].

Based on systematic kinetic measurements, Liu and co-workers show that the two catalysts exhibit similar H_2_O_2_ productivity in the H_2_+O_2_ reaction, and that the open-porosity TS-1 does not show a clear intrinsic advantage when free H_2_O_2_ is directly used in liquid-phase epoxidation. These results argue against a simple explanation based only on faster oxidant formation or better utilization of freely diffusing H_2_O_2_. Because the Au and Ti sites are located closer to each other, short-lived oxygen-containing intermediates, possibly including Au-bound hydroperoxy species, can be transferred more efficiently to neighboring Ti sites for the propylene epoxidation reaction, as shown in Fig. 1j. The catalyst therefore performs better not merely because of the enhancement in mass transport efficiency of gaseous reactants, but also because of the more efficient transfer of reactive intermediates.

Open porosity should not be regarded only as a diffusion modifier, but it should also be treated as a spatial design principle for multifunctional catalysis. Earlier discussions of hierarchical zeolites often focused on surface area, pore volume and accessibility [1], whereas this work places active site adjacency at the center of catalyst design. This catalyst design paradigm could be relevant far beyond Au/TS-1, especially for tandem systems in which one site generates a short-lived intermediate that must be rapidly captured by another. The next step is to promote this concept more quantitatively and more generally. Future studies should directly measure Au−Ti distance distributions under reaction conditions, decouple pore connectivity from metal loading and crystal size, and test whether similar open-porosity architectures can stabilize non-noble metals and translate these materials to other reactions. If so, open-porosity zeolites may evolve from a useful support modification into a broader platform for designing cooperative active sites in heterogeneous catalysis.

## References

[1] Chen L-H, Sun M-H, Wang Z et al. Chem Rev 2020; 120: 11194–294. 10.1021/acs.chemrev.0c00016 32915551

[2] Sun M-H, Zhou J, Hu Z-Y et al. Matter 2020; 3: 1226–45. 10.1016/j.matt.2020.07.016

[3] Zhao E-D, Chen Y, Xu J et al. Chem Synth 2025; 5: 63. 10.20517/cs.2024.180

[4] Lin D, Xu Y, Zheng X et al. AlChE J 2023; 69: e17999. 10.1002/aic.17999

[5] Feng X, Song Z, Liu Y et al. ACS Catal 2018; 8: 10649–57. 10.1021/acscatal.8b02836

[6] Liu Y, Zhao C, Sun B et al. Appl Catal A 2021; 624: 118329. 10.1016/j.apcata.2021.118329

[7] Xu J, Zhang Z, Yu D et al. Nano Res 2023; 16: 6278–89. 10.1007/s12274-023-5440-5

[8] He F, Lopez-Haro M, Chen T et al. Nat Synth 2026; 5: 761–74. 10.1038/s44160-025-00988-0

